# Transcriptomic changes in oxidative stress, immunity, and cancer pathways caused by cannabis vapor on alveolar epithelial cells

**DOI:** 10.1007/s10565-025-09997-3

**Published:** 2025-03-08

**Authors:** Emily T. Wilson, Percival Graham, David H. Eidelman, Carolyn J. Baglole

**Affiliations:** 1 Department of Pharmacology and Therapeutics, McGill University, Montreal, QC Canada; 2 Research Institute of the McGill University Health Centre, Montreal, QC H4A 3J1 Canada; 3 SCIREQ - Scientific Respiratory Equipment Inc, Montreal, Canada; 4 Department of Medicine, McGill University, Montreal, Canada

**Keywords:** Cannabis vapor, Air–liquid interface, Lung cancer, New approach methods

## Abstract

As legalization of cannabis increases worldwide, vaping cannabis is gaining popularity due to the belief that it is less harmful than smoking cannabis. However, the safety of cannabis vaping remains untested. To address this, we developed a physiologically relevant method for in vitro assessment of cannabis vapor on alveolar epithelial cell cultures. We compared the transcriptional response in three in vitro models of cannabis vapor exposure using A549 epithelial cells in submerged culture, pseudo-air liquid interface (ALI) culture, and ALI culture coupled with the expoCube™ advanced exposure system. Baseline gene expression in ALI-maintained A549 cells showed higher expression of type 2 alveolar epithelial (AEC2) genes related to surfactant production, ion movement, and barrier integrity. Acute exposure to cannabis vapor significantly affected gene expression in AEC2 cells belonging to pathways related to cancer, oxidative stress, and the immune response without being associated with a DNA damage response. This study identifies potential risks of cannabis vaping and underscores the need for further exploration into its respiratory health implications.

**Graphical Abstract:**

• Vaporizing cannabis is increasingly popular but remains largely untested.

• We used three in vitro models to assess the effects of cannabis vapor on alveolar epithelial cells.

• Cannabis vapor exposure alters pathways linked to cancer and metabolism, without causing DNA damage.

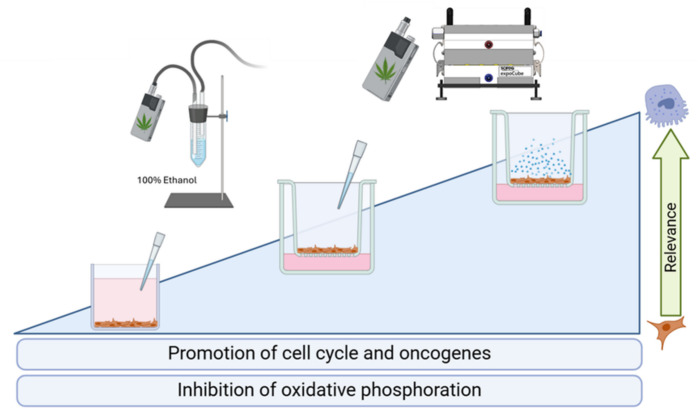

**Supplementary Information:**

The online version contains supplementary material available at 10.1007/s10565-025-09997-3.

## Introduction

*Cannabis sativa* (cannabis), a flowering plant, is commonly consumed by inhaling its smoke, which delivers biologically active compounds. The effects of cannabis are primarily attributed to cannabinoids like Δ^9^-tetrahydrocannabinol (Δ^9^-THC), which can interact with the endocannabinoid system (and other receptors) to modulate processes such as mood, pain perception, appetite, and immune responses (Banerjee et al. 2024; Nahas et al. 1999). Cannabinoids are released from the dry cannabis flower upon heating in a vaporizer or burning in the form of a joint, with the resultant vapor or smoke being inhaled by the user. Cannabis smoke contains pyrogenic compounds including carcinogens, mutagens, and teratogens which have the potential to cause adverse health outcomes (Graves et al. 2020). To reduce exposure to these pyrogenic compounds, various alternative methods for consuming cannabis have emerged, including products for vaporization, commonly known as cannavaping. Cannavaping encompasses devices like dab pens, which heat THC distillates or resins, as well as dry herb vaporizers that use dried cannabis flower as the input material similar to traditional cannabis joints (Aston et al. 2019; Varlet et al. 2016). Dry herb vaporizers heat the cannabis flower without burning it, releasing cannabinoids while reducing exposure to harmful byproducts (Lanz et al. 2016). Although research has explored the effects of cannabis vapes using dab pens and aerosols (Grotenhermen 2001; Vulfsons et al. 2019), the impacts of dry herb vaporizers on the respiratory system remain largely unstudied, despite their rising popularity.

Pulmonary epithelial cells are among the first cells to encounter inhaled substances. In vitro approaches provide a convenient means to directly investigate the effects of cannabis vapor on lung epithelial cell function, and further enable high-throughput mechanistic insights that are relevant to human biology. Typically, cell culture is performed using a submerged method, where cells are grown on plastic and exposed apically to media. However, epithelial cells lining the respiratory system are unique because in vivo, the apical surface is in contact with air, a feature of their biology which submerged cultures fail to replicate. An alternative is to culture lung epithelial cells at ALI. Here, respiratory epithelial cells are grown on Transwell permeable supports that allow for the apical surface to be exposed to air. Lung epithelial cells cultured at ALI can polarize, produce surfactant, and form junctions (Varlet et al. 2016; Wu et al. 2017), making this a model that captures many physiological properties of the respiratory epithelium.

Another consideration for in vitro studies involving inhalational exposures is the physiochemical properties of the compounds contained therein. For cannabis, this includes cannabinoids which are hydrophobic and thus do not dissolve into aqueous media (Kaur et al. 2022). To address this, solvents such as ethanol are used (Adekola et al. 2022) to permit both hydrophobic and hydrophilic particles to be dissolved into the cell culture media. Some studies have dissolved treatment compounds in media and then applied these solutions to the apical surface of cells kept under ALI conditions, a technique referred to as pseudo-ALI (Kaur et al. 2022). However, compounds may agglomerate and/or interact with the media, thereby limiting their bioavailability (Lenz et al. 2013; Upadhyay and Palmberg 2018). The recent development of advanced exposure systems addresses this limitation by allowing for the delivery of aerosols and particle suspensions to the apical surface of cells at ALI. In this study, we compared the biological and toxicological response of alveolar epithelial cells to cannabis vapor using submerged, pseudo-ALI, and ALI models. Our findings not only illuminate the complexities inherent to studying the effects of cannabis vapor exposure on respiratory cell biology but also pave the way for physiologically relevant research in the field of inhalation toxicology. Most importantly, these results demonstrate for the first time that cannabis vapor elicits significant changes in cellular pathways related to cancer, oxidative stress, and immunity in alveolar epithelial cells, raising concerns for habitual users and highlighting the urgent need for additional research in light of increasing legalization.

## Methods

### Submerged cell culture

The human adenocarcinoma A549 cell line was purchased from American Type Culture Collection (ATCC # CCL-185, Manassas, VA, USA). Cells were cultured in Dulbecco's Modified Eagle Medium (DMEM) supplemented with 10% fetal bovine serum, 1% Glutamax, 1% antibiotic–antimycotic, and 0.1% gentamycin and maintained at 37 °C with 5% CO_2_. For experiments using submerged cultures, cells were seeded into 6 well plates at a density of 2 × 10^5^ cells/ml. Cells were grown to 80% confluency before exposure.

### ALI cell culture

Membranes of Millicell inserts (0.4-µm pore, 12 mm inserts, polycarbonate, Millipore Sigma, Burlington, MA, USA) were pre-coated with 70 μg of collagen type IV from human placenta (Millipore Sigma, Burlington, MA, USA). Single cell suspensions of A549 cells were seeded on apical surfaces of collagen-pre-coated membranes with a density of 2.5 × 10^5^ cells per well using prepared DMEM described above. The culture medium on the apical side was removed 24 h after the seeding to establish the ALI condition. The ALI cultured cells were refreshed with DMEM in the bottom of the insert at two-day intervals and allowed to differentiate for 14 days (Wu et al. 2017).

### Cannabis selection and preparation

Cannabis was purchased from the *Société québécoise du cannabis* (SQDC) online store (Montreal, QC, Canada). Whole flower cannabis that was selected for purchase was Indica-THC dominant containing 16–22% THC and 0–0.1% CBD (Lot #688,083,002,311). Dried cannabis flower was ground with a plastic grinder. A DaVinci MIQRO dry cannabis vaporizer was used to vaporize the cannabis in 0.25 g increments at 188° C-199° C.

### Generation of cannabis vapor extract (CaVE)

CaVE was created using the inExpose™ (SCIREQ, Montreal, QC, Canada) system equipped with an electronic device attachment to generate cannabis vapor with a Davinci MIQRO vaporizer. Cannabis vapor was generated using the inExpose™ at 4 puffs/min with a puff volume of 70 ml lasting 4 s. This vapor was bubbled through 10 ml ethanol for 10 min, replacing with 0.25 g of fresh cannabis every 5 min (0.5 g of cannabis total) as we have previously described (Aloufi et al. 2022). Three- 10 ml CaVE preparations were generated and pooled together. The extract was stored at −80° C.

### expoCube™ exposures

Cells cultured at ALI in four 12-well plates were placed in the expoCube™ (SCIREQ, Montreal, QC, Canada) which was prewarmed to establish a thermophoretic gradient from 35 °C on the bottom unit to 50 °C at the top unit. Humidity was set to 60–80%. Thirty-minute exposures were performed using the DaVinci MIQRO to generate the cannabis vapor at 4 puffs/min with a puff volume of 70 ml lasting 4 s. During the 30-min treatment, the DaVinci MIQRO dry herb vaporizer was packed with 0.25 g of cannabis at a time and ran for five minutes before switching out cannabis material. The treatment particle suspension was humidified using an Aeroneb system and fed into an isokinetic sampler (Supp. Figure 1a). Cells were exposed to the humidified cannabis vapor within the exposure chamber at a controlled distance over a temperature gradient to increase deposition (Supp. Figure 1b). Exposures included the treatment (cannabis vapor), a vehicle control (humidified air only), as well as a negative control (not exposed to airflow) (Supp. Figure 1b-c). All expoCube™ treatments were performed on the same day. After treatment, cells were incubated with the deposited cannabis vapor for 24 h.

### Cytotoxicity

Cytotoxicity was assessed by lactate dehydrogenase (LDH) release from cells using an LDH kit (No. 11644793001, Roche Diagnostics, Mannheim, Germany). After 24 h of treatment, 100 µl of media was taken for testing. ALI and pseudo-ALI media samples were diluted 1:20 in serum free DMEM. One hundred µl of sample was incubated with 100 µl of reaction mixture for 25 min. Absorbance was measured at a wavelength of 490 nm with a reference wavelength of 600 nm. Measurements were corrected for background and normalized to the 2% triton-X treated cells. Cytotoxicity was defined as a significant increase in LDH release that exceeds 10% when compared to the negative control.

### Experimental design

We compared three models of cannabis vapor exposure using A549 cells from passage 12–18 (Supp. Figure 2). The submerged model consisted of A549 cells grown in a traditional submerged culture and treated with CaVE, ethanol as the vehicle control, or DMEM only. The pseudo-ALI model consisted of A549 cells grown at ALI for 2 weeks before being treated with CaVE in 100 µl of DMEM, ethanol vehicle in DMEM, or only DMEM to the apical compartment. Lastly, the ALI model consisted of A549 cells grown at ALI for 2 weeks before being exposed to cannabis vapor through the expoCube™ exposure system for 30 min. ALI expoCube™ exposures were performed and 24 h after exposure, the concentration of Δ^9^-THC in the basolateral compartment was analyzed by a direct competitive THC Forensic ELISA kit (NEOGEN, Lansing, MI, USA) according to manufacturer’s instructions and quantified to a standard curve (Aloufi et al. 2022). The absorbance was read at 450 nm by the Tecan Infinite M200 plate reader (TECAN, Männedorf, Switzerland). All models were exposed for 24 h before sample collection.

### RNA sequencing (RNA-seq)

Total RNA was isolated from cells using the Aurum™ Total RNA Kit (Bio-Rad, Montreal, QC, Canada). The quality and quantity of RNA was assessed using the Tecan Infinite M200 plate reader (TECAN, Männedorf, Switzerland). Total RNA underwent mRNA purification using poly-T oligo-attached magnetic beads, followed by cDNA synthesis in two stages- first strand synthesis with random hexamer primers and second strand synthesis. The resultant library quality and quantity were assessed using Qubit, real-time PCR, and a bioanalyzer for size distribution. These prepared libraries were then pooled for sequencing on Illumina platforms using a PE150 strategy (Novogene Corp., Sacramento, CA, USA). The raw fastq format data underwent initial processing with Novogene Perl scripts to remove adapters and low-quality reads, resulting in clean data. The quality of this data was assessed based on Q20, Q30, and GC content. The hg38 reference genome and annotations were sourced from the genome database. HISAT2 v2.0.5 was used for genome indexing and aligning paired-end reads. Gene expression levels were quantified using featureCounts v1.5.0-p3 by counting mapped reads, and normalized counts were calculated for each gene.

### Differential gene expression analysis and gene set enrichment analysis (GSEA)

Differential gene expression between two conditions (each with four biological replicates) was analyzed using the DESeq2 R package, based on the negative binomial distribution model (Love et al. 2014). GSEA was performed using the clusterProfiler, msigdbr, and org.Hs.eg.db R packages with GSEA version 4.3.3 to examine hallmark pathways associated with differential gene expression changes (Subramanian et al. 2005). Differentially expressed genes (DEGs), defined by *p* < 0.05, were ranked by log2 fold-change. For pathway selection, we used the hallmark pathways from MSigDB (H collection), specifically curated for *Homo sapiens*. Significance was set at *p* < 0.05 to identify significantly enriched pathways, with the output generating normalized enrichment scores and significance values for each pathway. To visualize the GSEA results, dotplots and cnetplots of top three activated and suppressed pathways for each comparison were generated (Supp. File 1). Bar graphs and heatmaps of z-scores and log2 fold-changes were generated using GraphPad Prism (v10.2.1).

### Genotoxicity prediction analysis

Classification of cannabis vapor as DNA damage inducing (DDI) or Non-DDI (NDDI) was performed by comparing gene expression signatures of cells treated with cannabis vapor versus vehicle control with those in the TGx-DDI database. Compounds with a gene expression pattern similar to known DDI profiles were classified as DNA-damaging, whereas those aligning with non-DNA damage signatures were classified as NDDI (Li et al. 2019).

## Results

### CaVE causes inflammation and cell cycle arrest in A549 cells under submerged conditions

**Fig. 1:**
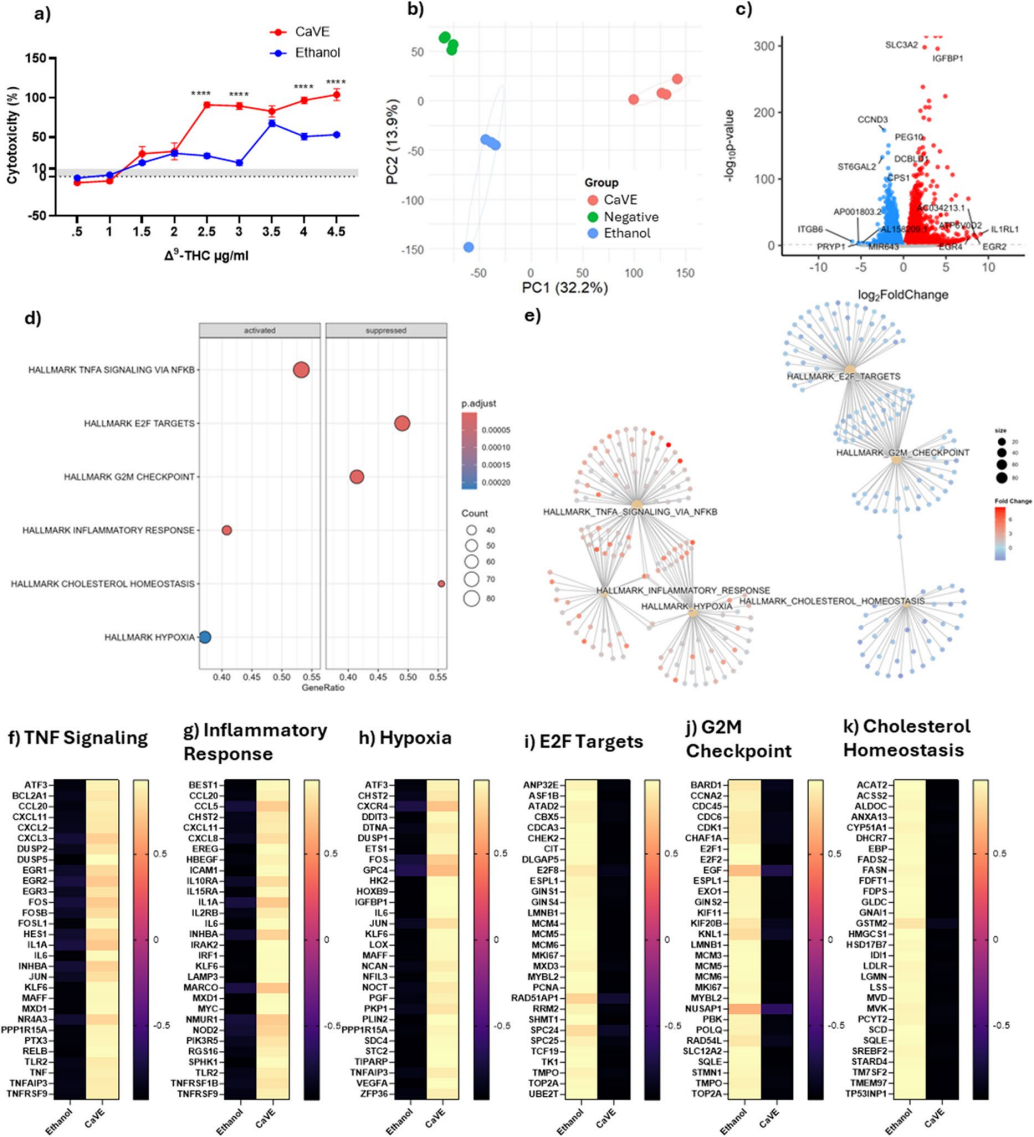
CaVE causes inflammation in A549 cells cultured under submerged conditions. (**a**) Cytotoxicity of CaVE or volume-equivalent vehicle control across a dose range of 0–4.5 µg ${\Delta }^{9}$-THC/ml. (**b**) PCA of cell cultures treated with CaVE, ethanol vehicle control, or negative control (**c**) Volcano plot showing the transcriptional response of CaVE versus vehicle control in submerged A549 cells. (**d**) GSEA of pathways significantly enriched in response to CaVE. (**e**) Cnetplot illustrating gene similarities and overlaps among the top enriched pathways. (**f**–**k**) Heatmaps with z-scores of genes involved in the top enriched pathways in response to CaVE, including TNF Signaling (**f**), Inflammatory Response (**g**), Hypoxia (**h**), E2F Targets (**i**), G2M Checkpoint (**j**), and Cholesterol Homeostasis (**k**)

Cannabis vaporizers are novel devices and their effects on the pulmonary system remain poorly characterized. To investigate the impacts of cannabis vapor on human lung epithelial cells, we treated A549 cells in a submerged model with CaVE at doses ranging from 0–4.5 µg/ml of Δ^9^-THC or equivalent volumes of ethanol vehicle control. CaVE caused significant cytotoxicity at a dose of 2.5 µg/ml (Fig. 1a). Based on this result, we selected 2 µg/ml for further transcriptomic analysis using RNA sequencing. Principal Component Analysis (PCA) showed clear separation between the CaVE, the vehicle, and the negative control groups (Fig. 1b). The first principal component (PC1) explained 32.2% of the variance, while the second component (PC2) explained 13.9%, indicating that the transcriptional changes were largely driven by treatment with CaVE.

We then assessed DEGs affected by CaVE (Fig. 1c, Supp. Table 1). Some of the most upregulated genes included *EGR2* and *EGR4*, which are involved in epithelial cell proliferation and differentiation (Kumbrink et al. 2010). To understand the pathways these DEGs contribute to, we performed GSEA (Fig. 1d, Supp. Table 2). The top three upregulated pathways included TNF signaling via NF-κB, inflammatory response, and hypoxia. The top three downregulated pathways were E2F targets, G2M checkpoint, and cholesterol homeostasis. Clustering of these pathways was also performed using cnetplots to visualize overlaps in genes (Fig. 1e). All upregulated pathways clustered together. The downregulated pathways E2F targets and the G2M checkpoint clustered together, while the pathways associated with cholesterol homeostasis remained distinct. Clustering indicates an overlap of genes contributing to these pathways, signifying that in general these pathways are interrelated.

The most upregulated genes in response to cannabis vapor were related to inflammation (Fig. 1f-g) and included chemokines such as those from the *CXCL*, *CCL*, interleukins, and *TNF* families, suggesting induction of inflammation in the epithelial cells. Genes in the hypoxia pathway were also upregulated by CaVE (Fig. 1h), including DNA damage response genes such as *DDIT3* and *TIPARP*, which are involved in cellular stress adaptation and repair (Hutin et al. 2021; Osman et al. 2023). Additionally, CaVE upregulated *FOS* and *JUN* which are key components of the AP-1 complex, a transcriptional factor that regulates cell proliferation, differentiation, stress response, and DNA damage repair mechanisms (Rauscher et al. 1988). The downregulation of cell cycle pathways, including E2F targets and the G2M checkpoint, indicates an impairment in cell proliferation (Fig. 1i-j). Lastly, genes involved in cholesterol homeostasis were downregulated (Fig. 1k). These genes play a role in cholesterol biosynthesis, fatty acid and lipid metabolism, and cholesterol efflux, all of which are critical in the production of surfactant by AEC2 cells. Together these results show that CaVE exposure induces significant transcriptional changes in submerged A549 cells, characterized by inflammation, hypoxia-related responses, and activation of DNA damage repair mechanisms, while simultaneously downregulating those associated with cell cycle progression and homeostasis.

### Alveolar epithelial cells cultured under pseudo-ALI and ALI conditions display enhanced AEC2-associated gene expression

**Fig. 2:**
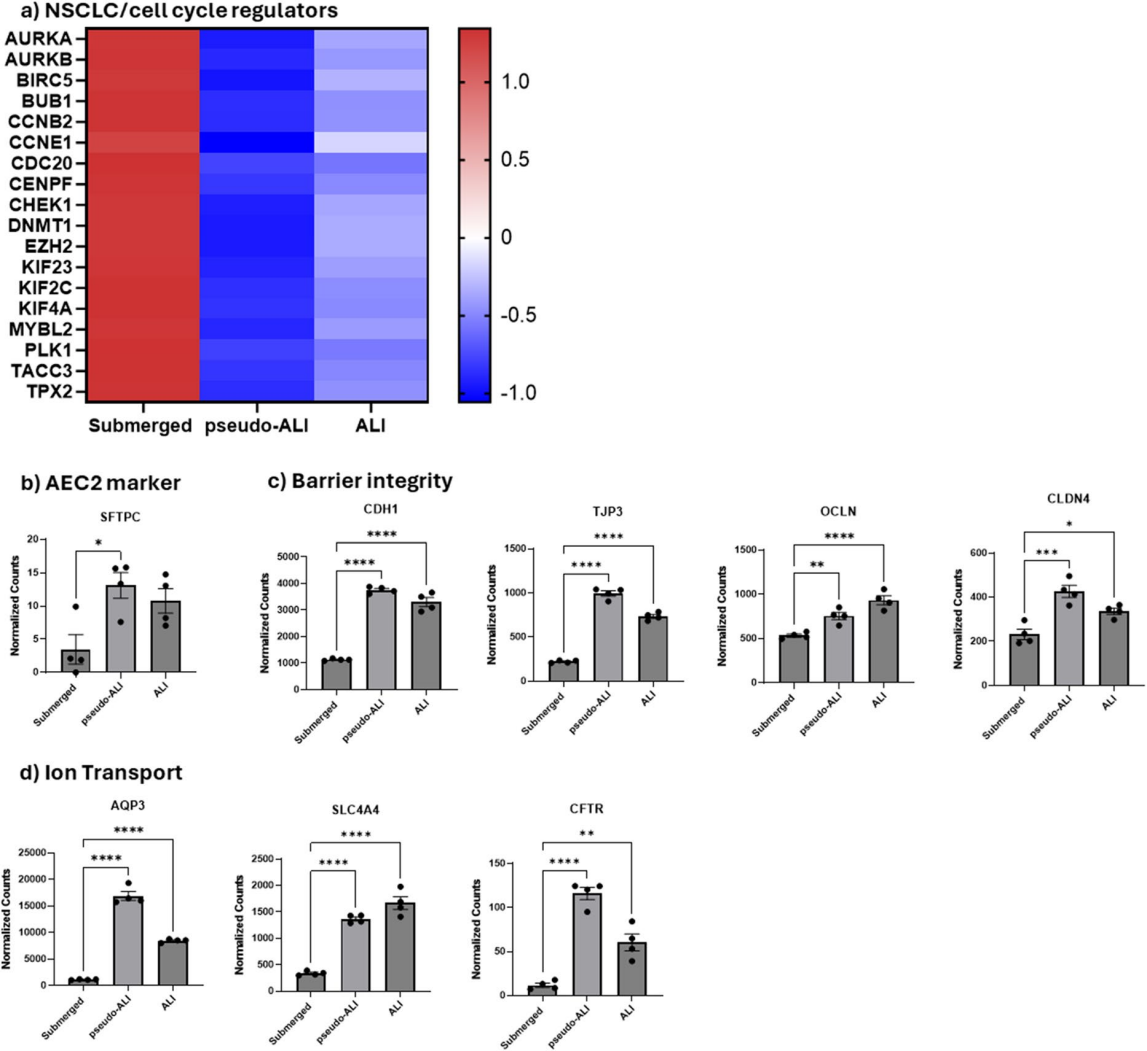
Cells cultured at ALI show decreased markers of NSCLC and increased markers of alveolar epithelial cells. (a) Heatmap displaying z-scores of NSCLC markers and cell cycle regulators across submerged, pseudo-ALI, and ALI culture conditions. (b) Expression of the AEC2 marker *SFTPC* is elevated in pseudo-ALI and ALI cultures. (c-d) Culturing cells at ALI enhances the expression of genes associated with alveolar functions, including (c) barrier integrity markers *CDH1*, *TJP3*, *OCLN*, and *CLDN4* and (d) ion transport-related genes *AQP3*, *SLC4A4*, and *CFTR*. Statistical analysis was performed using two-way ANOVA followed by Dunnett’s test (*p < 0.05, **p < 0.01, ***p < 0.001, ****p < 0.0001)

A549 cells are a widely used AEC2 cell model. One of the limitations of this cell line is that A549 cells are derived from non-small cell lung cancer (NSCLC) and thus exhibit cancer-like characteristics, including uncontrolled proliferation. A major advantage of ALI cultures is their ability to allow cells to reach a non-proliferative, terminal state. To test this, we assessed markers of NSCLC in cells cultured under submerged, pseudo- and ALI conditions (Fig. 2a). We observed a decrease in NSCLC and cell cycle-associated gene expression when cells were cultured at pseudo-ALI and ALI. For example, the expression of *AURKA*, a gene frequently overexpressed in NSCLC and linked to uncontrolled cell proliferation, was reduced in pseudo- and ALI conditions (Zheng et al. 2018). This suggests that culturing A549 cells at ALI brings their transcriptional expression closer to that of AEC2 cells.

Major functions of AEC2 cells include surfactant production, fluid regulation, and barrier integrity (Ballard et al. 2010). To determine whether expected differentiation was taking place in our model, we interrogated our RNA-seq data to evaluate the impact of culture conditions on the expression of key genes in unexposed cells. First, we compared the expression of genes important for surfactant protein production. *SFTPC*, a marker of AEC2 cells, was increased in ALI and pseudo-ALI cell culture models compared to submerged cell cultures (Fig. 2b). We next assessed genes related to epithelial barrier integrity, which in AEC2 cells is made up of proteins for tight junctions, cadherins, and claudins (Díaz-Coránguez et al. 2019). Genes of proteins involved in cell-to-cell junctions and cell-to-cell contact such as ZO-1 (*TJP3)*, E-Cadherin (*CDH1)*, occludin (*OCLN*), and CLDN4 (*CLDN4)* were also expressed at higher levels in pseudo-ALI and ALI cultures than submerged cells (Fig. 2c). Lastly, genes associated with water transport and ion channels (Ruaro et al. 2021), including *CFTR*, *AQP3*, and *SLC4A4* also had increased expression in pseudo-ALI and ALI cultures (Fig. 2d). It is noteworthy that between cells cultured at pseudo-ALI versus ALI, there was little difference in the expression of genes involved in surfactant production, water and ion channels or cell–cell junctions. Thus, pseudo- and ALI cultures promote a transcriptomic response more closely mirroring the phenotype of AEC2.

### CaVE promotes cell cycle progression and suppresses oxidative phosphorylation in A549 pseudo-ALI cultures

**Fig. 3:**
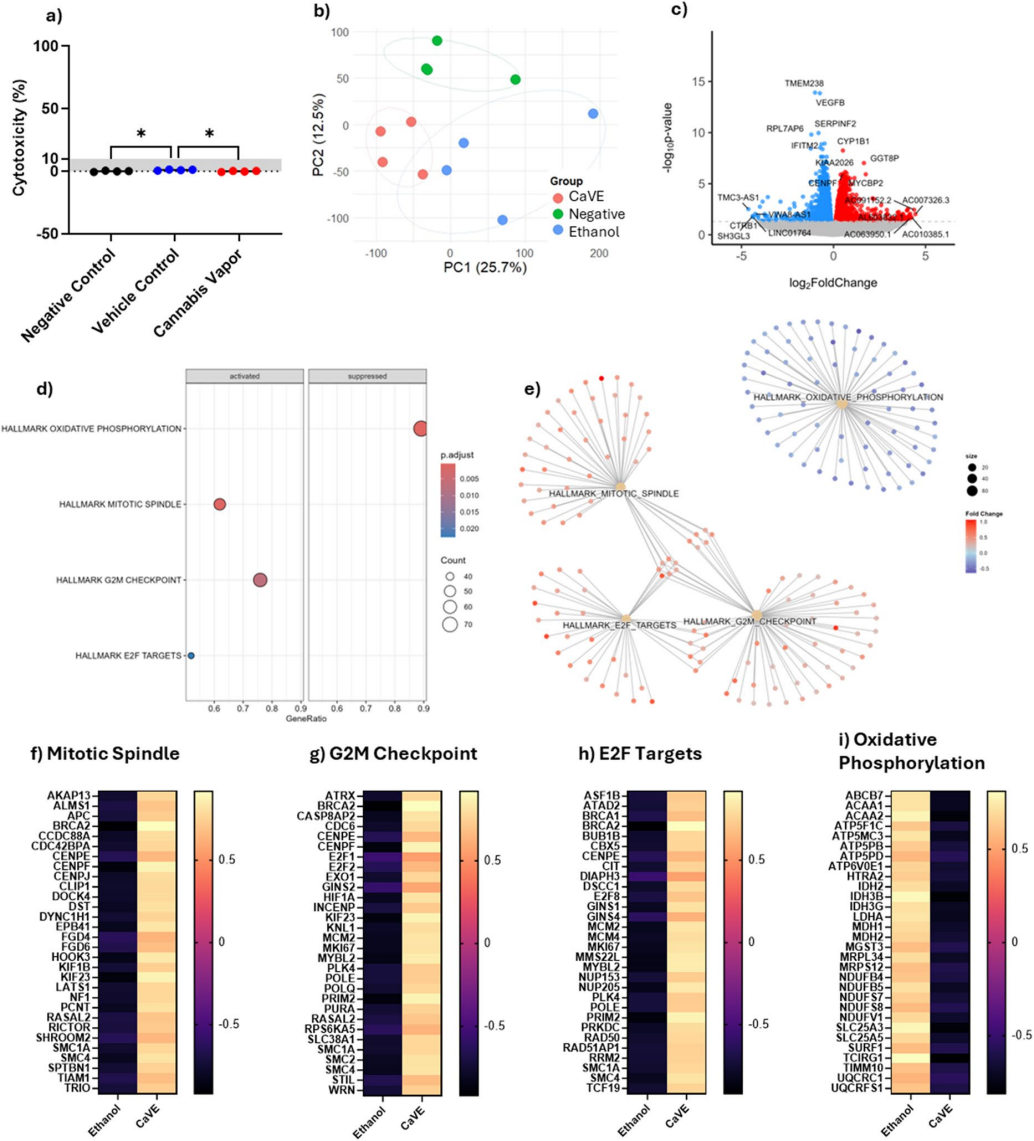
CaVE alters metabolism and cell growth pathways in pseudo-ALI cultures. (a) Cytotoxicity of CaVE, volume-equivalent ethanol control and negative control in pseudo-ALI cultures. (b) PCA of transcriptional data from pseudo-ALI A549 cultures treated with CaVE, vehicle control, or negative control. (c) Volcano plot of the transcriptional response of CaVE versus vehicle control in pseudo-ALI A549 cells. (d) GSEA of significantly enriched pathways in response to CaVE. (e) Cnetplot showing gene similarities and overlaps among the top enriched pathways. (f–i) Heatmaps with z-scores of genes involved in the top enriched pathways, including Mitotic Spindle (f), G2M Checkpoint (g), E2F Targets (h), and Oxidative Phosphorylation (i)

We next tested the impacts of CaVE in A549 cells cultured at ALI with CaVE diluted in DMEM to a final concentration of 2 µg/ml Δ^9^-THC, equivalent volume of ethanol in DMEM (vehicle control), or DMEM alone (negative control). The vehicle elicited a minimal cytotoxic response of 1.1%, and CaVE showed no significant impact on cytotoxicity (Fig. 3a). PCA of the RNA-seq data showed separation of the negative control group from both CaVE-treated and the ethanol-vehicle control groups (Fig. 3b). While CaVE and vehicle control cells clustered separately, they were close in proximity, suggesting that vehicle effect in the ALI model may partially account for some of the effects of cannabis vapor in this model.

We next identified the DEGs between CaVE exposed and vehicle control cells (Fig. 3c, Supp. Table 3). Notable upregulated genes included *CYP1B1*, a xenobiotic metabolizing enzyme frequently overexpressed in cancer (Murray et al. 1997). GSEA pathway analysis of DEGs revealed the upregulation of key pathways involved in cell cycle progression, including the mitotic spindle, G2M checkpoint, and E2F targets (Fig. 3d, Supp Table 4). These pathways are critical for proper mitosis, DNA replication, and checkpoint regulation. In contrast, oxidative phosphorylation was significantly suppressed by CaVE. Clustering analysis using cnetplot showed that the upregulated pathways clustered together, with overlapping genes driving their enrichment, indicating that they are interrelated. In contrast, the oxidative phosphorylation pathway clustered separately (Fig. 3e). Genes in the upregulated cell cycle pathways (Fig. 3f-h) included oncogenes such as *BRCA2*, which is involved in homologous recombination-mediated DNA repair (Abaji et al. 2005), and E2F family members, which drive cell cycle progression through S-phase entry (Pennycook et al. 2020). There was downregulation in oxidative phosphorylation after treatment with CaVE (Fig. 3i). This included a decrease in genes associated with electron transport chain (ETC) complexes, including complex I ubiquinone oxidoreductase (*NDUF* gene family), complex III cytochrome c reductase (*UQCR* gene family), and complex V ATP synthase (*ATP5* gene family) (Fig. 3i). Together, these findings demonstrate that CaVE exposure in A549 cells cultured at ALI may promote cell cycle progression while simultaneously suppressing oxidative phosphorylation.

### Cannabis vapor promotes cell growth and oncogenic non-coding RNA expression in A549 cells cultured at ALI

**Fig. 4:**
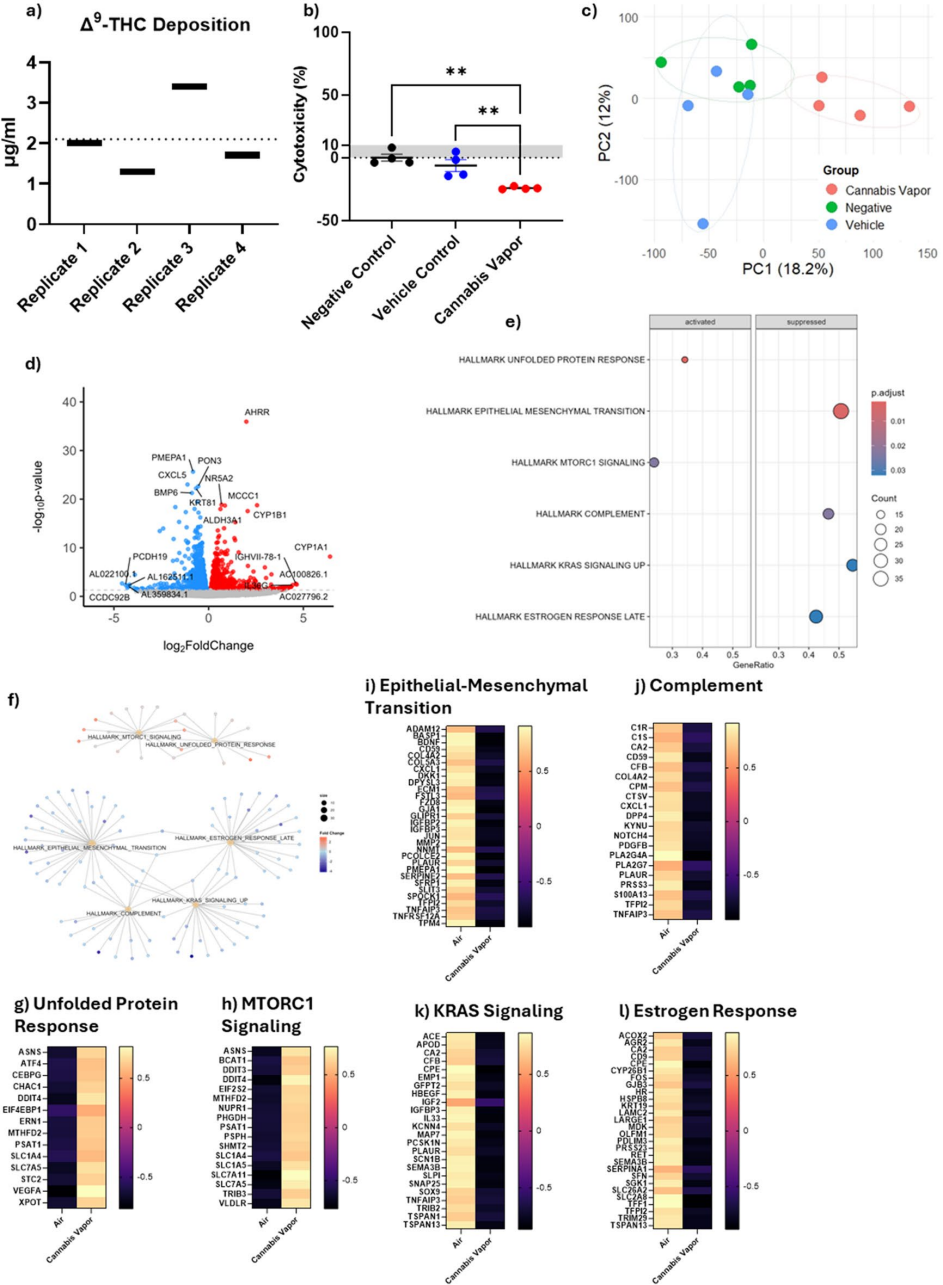
Cannabis vapor alters metabolism and cell growth pathways in ALI cultures. (a) Cannabis vapor deposition in cell culture. (b) Cytotoxicity of cannabis vapor or air vehicle control compared to negative control. (c) PCA of transcriptional data from A549 cells treated with cannabis vapor, vehicle control, or negative control. (d) Volcano plot of the transcriptional response of cannabis vapor versus vehicle control in ALI A549 cells. (e) GSEA of significantly enriched pathways in response to cannabis vapor exposure. (f) Cnetplot showing gene similarities and overlaps among the top enriched pathways. (g–l) Heatmaps with z-scores of genes involved in the top enriched pathways, including Unfolded Protein Response (g), MTORC1 Signaling (h), Epithelial-Mesenchymal Transition (i), Complement (j), KRAS Signaling (k), and Estrogen Response (l)

Given that ALI cultures exhibited evidence of AEC2-like gene expression, we utilized the RNA-seq analysis from A549 cells in this model to better understand the effect of whole cannabis vapor. Using an advanced exposure system, we achieved an average cannabis vapor deposition of 2.1 µg Δ^9^-THC/ml, with values ranging from 1.3 to 3.4 µg/ml (Fig. 4a). At this concentration, there was no increase in cytotoxicity compared to controls (Fig. 4b). PCA demonstrates distinct clustering patterns of alveolar epithelial cells following exposure to cannabis vapor, with some overlap between the vehicle and negative control groups (Fig. 4c). This indicates a lack of vehicle effect. We next identified DEGs following cannabis vapor exposure (Fig. 4d, Supp. Table 5). Notable upregulated genes included *AHRR*, *CYP1B1*, and *CYP1A1*, genes which are regulated by the aryl hydrocarbon receptor (AhR) (Peres et al. 2017). Activation of the AhR is a well-documented response to exposure to toxic substances, suggesting that cannabis vapor triggers a detoxification response. GSEA pathway analysis of DEGs revealed that cannabis vapor significantly upregulated pathways related to cell metabolism and growth, and downregulated pathways involved in migration and inflammation (Fig. 4e, Supp Table 6). Cnetplot clustering showed some overlap in both downregulated and upregulated pathways (Fig. 4f). Upregulated pathways involved in cell metabolism included the unfolded protein response and mTORC1 signaling pathway (Fig. 4g-h), suggesting that cannabis vapor promotes cellular adaptation to stress. Conversely, epithelial-to-mesenchymal transition (EMT), complement activation, KRAS signaling, and estrogen receptor signaling pathways were downregulated (Fig. 4i-l) together indicating a dysregulation of cell growth and metabolism pathways.

We also found significant changes in non-coding RNAs (ncRNAs) that play critical roles in gene expression regulation (Table 1). We identified 18 long non-coding RNAs (lncRNAs) that were upregulated and 10 that were downregulated. Notably, among the upregulated lncRNAs was LINC00115, which is highly expressed in tumor tissues and cells (Wu et al. 2022). On the other hand, DIO3OS was downregulated; this lncRNA is known to be downregulated in NSCLC (Zhang et al. 2021). Moreover, our results also showed that three microRNAs (miRNAs) were significantly upregulated. Of the upregulated miRNAs, both MIR27b and MIR421 are identified oncogenes contributing to progression of various cancers (Dong et al. 2020; Hannafon et al. 2019). Thus, acute cannabis vapor exposure promoted pathways associated with cancer and cell growth, and markedly increased oncogenic ncRNAs. Taken together, these results demonstrate that acute cannabis vapor exposure promotes pathways associated with cellular stress, metabolism, and growth, while simultaneously increasing the expression of oncogenic ncRNAs. This dysregulation highlights the potential of cannabis vapor to contribute to pro-cancerous processes in the lung epithelium.

**Table 1:** Expression of ncRNA in A549 cells exposed to vaporized cannabis

lncRNA		miRNA	
Upregulated	Downregulated	Upregulated	Downregulated
AL645933.2	AL162511.1	MIR27B	
LINC00410	AL359834.1	MIR3936	
ZNF295-AS1	BLACAT1	MIR421	
AC005330.1	AC004816.2		
LINC01730	CU639417.4		
AC092119.2	FAM157C		
LINC00115	AC009061.1		
AC244197.2	AC084757.3		
AC005838.2	DIO3OS		
LINC00562	AL121790.2		
AC008507.2			
AC087491.1			
AC106017.2			
AC126696.3			
AC141002.1			
AL136115.1			
AC008752.1			
AC100826.1			

### Cannabis vapor does not exhibit a genotoxic mechanism of action

**Fig. 5:**
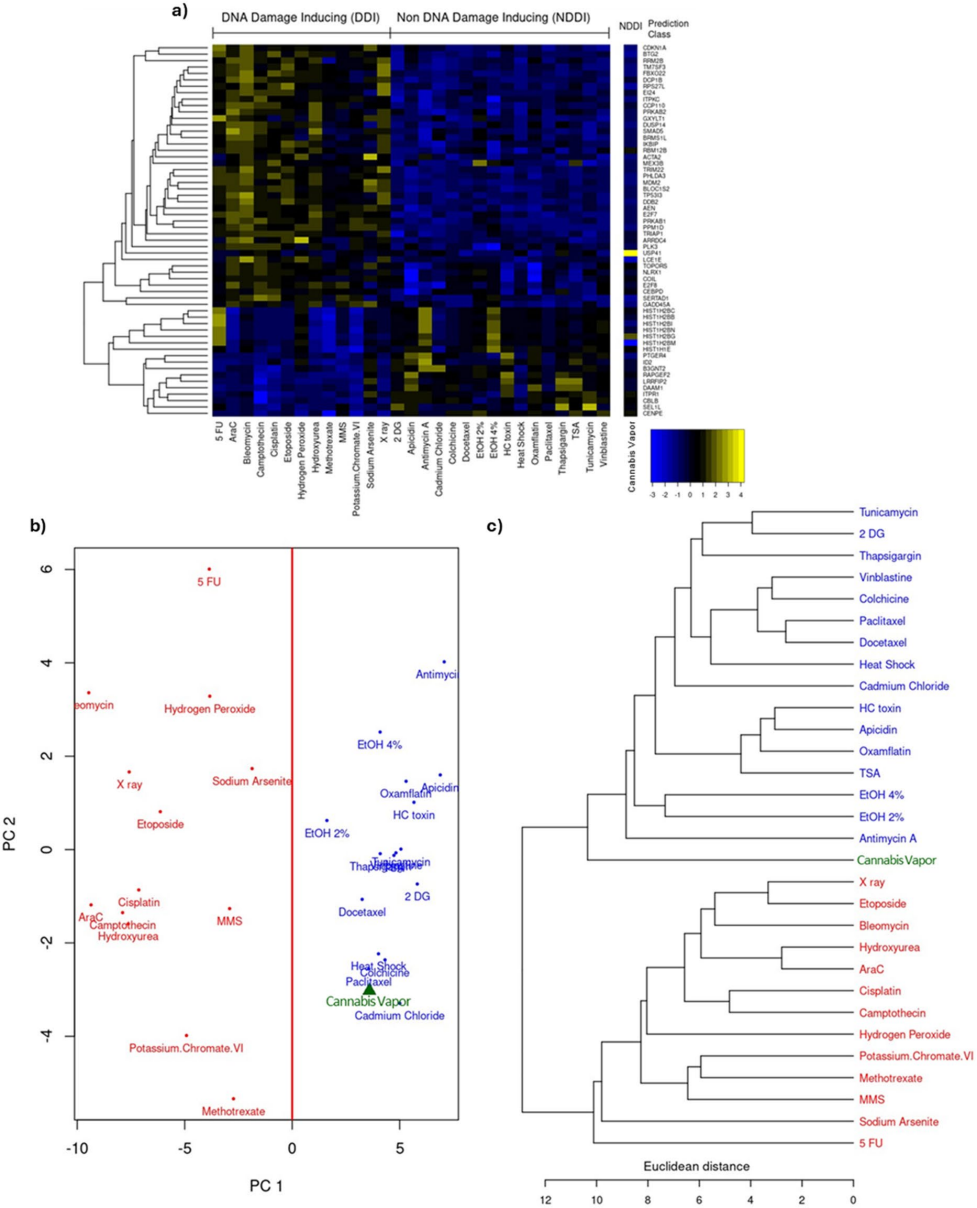
Cannabis vapor does not elicit genotoxic effects in alveolar epithelial cells. (a) Heatmap, (b) PCA, and (c) hierarchical clustering using Euclidean distances with average linkage of transcriptome profiling data illustrating co-expressed sets of genes associated with genotoxic and non-genotoxic compounds

Because we observed significant changes in cancer-related pathways, we next sought to understand the potential genotoxicity of cannabis vapor exposure. Genotoxicity is one of the primary mechanisms through which carcinogenic materials induce oncogenesis. To assess the genotoxic potential of cannabis vapor, we used the TGx-DDI biomarker for DNA damage classification (Li et al. 2019). This methodology was designed for identifying DDIs and operates by analyzing stress response-related gene expression alterations using an established gene expression database against which new compounds are compared, thereby categorizing them as DDI or NDDI (Li et al. 2019). In Fig. 5a, the heatmap shows the comparison of the transcriptomic response of A549 cells to cannabis vapor with the transcriptomic signatures of known DDI and NDDI compounds. The compound prediction class for cannabis vapor based on this analysis is classified as NDDI. PCA and hierarchical clustering illustrate the degree of similarity between cannabis vapor and the compounds of the database (Fig. 5b-c) whereby cannabis vapor clusters on the NDDI side of the PCA plot and hierarchical cluster. Overall, these results show that vaporized cannabis affects key cellular pathways related to cancer in AEC2 cells through a non-genotoxic mechanism, highlighting the need for further research on the potential health implications for habitual users.

### Cannabis vapor elicits transcriptional changes in pathways regulating cell division, metabolism, and migration across all models

**Fig. 6:**
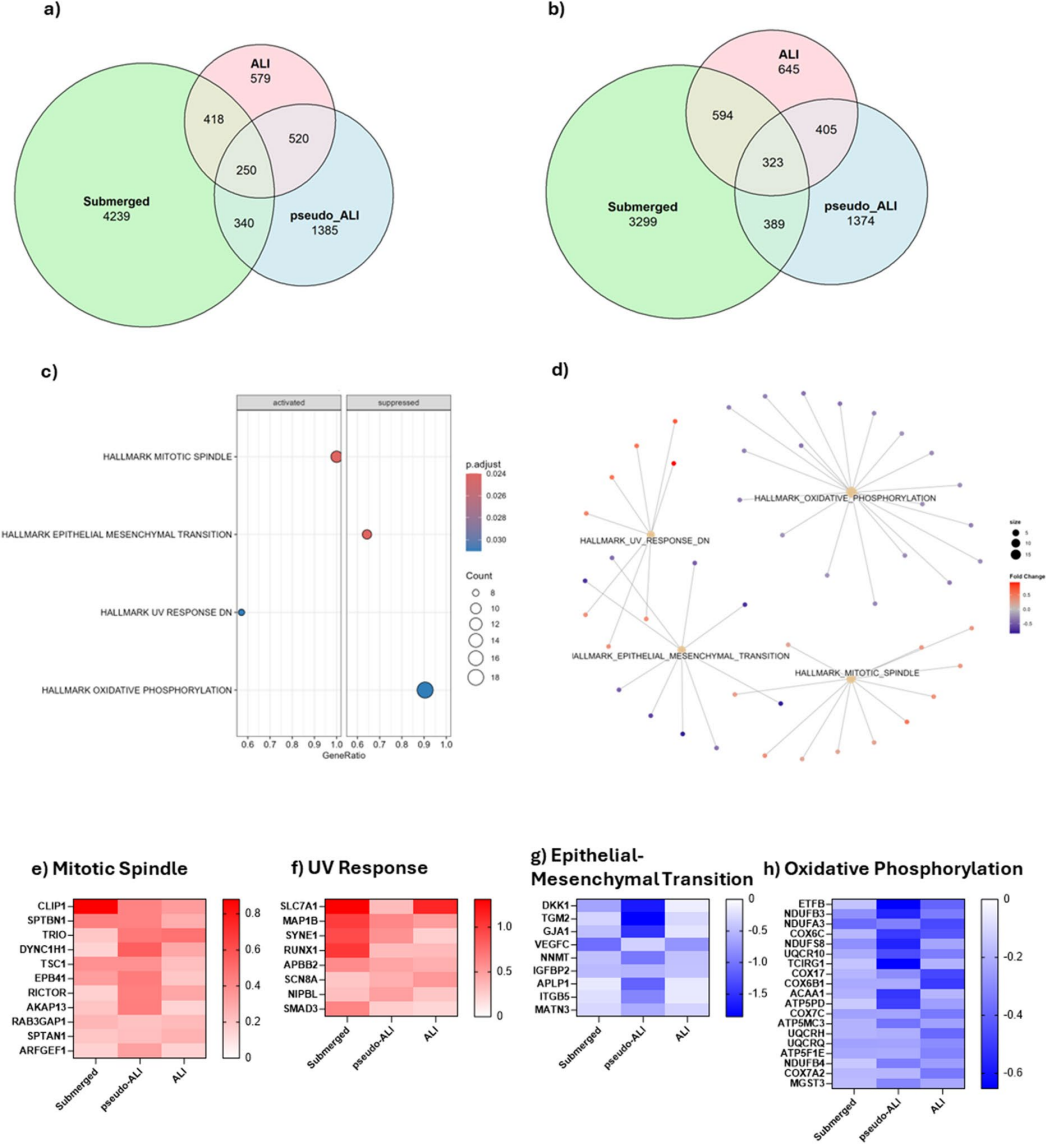
Transcriptional changes in A549 cells in response to cannabis vapor exposure across submerged, pseudo-ALI, and ALI models. (a) Venn diagram showing DEGs upregulated by cannabis vapor across models. (b) Venn diagram showing DEGs downregulated by cannabis across models. (c) GSEA of DEGs common to all models. (d) Cnetplot of enriched pathways. (e–h) Heatmaps displaying log2 fold change values of DEGs of cannabis vapor versus vehicle control in each model related to (e) Mitotic Spindle, (f) UV Response, (g) Epithelial-Mesenchymal Transition, and (h) Oxidative Phosphorylation pathways

Finally, we evaluated the transcriptional profile of A549 cells in response to cannabis vapor across all three models to visualize conserved pathways. Cannabis vapor significantly altered transcriptional activity compared to vehicle control in all models, with the submerged model showing the most changes for both upregulated (Fig. 6a, Supp. Table 7) and downregulated genes (Fig. 6b, Supp. Table 8). To understand what impacts of cannabis vapor are conserved regardless of model, we next ran GSEA on genes that were upregulated or downregulated in all three models. GSEA identified activation of the mitotic spindle and UV response pathways, along with suppression of EMT and oxidative phosphorylation (Fig. 6c, Supp. Table 9). The cnetplot showed that these pathways are independent, with no overlapping genes (Fig. 6d).

Genes in the mitotic spindle pathway and UV response pathway (Fig. 6e-f) are critical for cell growth signaling through the mTOR pathway as well as cytoskeletal organization, migration, and division. In the EMT pathway (Fig. 6g), there was downregulation of genes critical for cell adhesion as well as Wnt signaling inhibitor *DKK1* (Wall et al. 2020). Lastly, the suppression of oxidative phosphorylation genes (Fig. 6h), encoding components of the mitochondrial ETC, indicate a shift from oxidative phosphorylation to glycolysis. Overall, these results suggest that, regardless of exposure and/or culture condition, cannabis vapor promotes changes in key pathways regulating cell division, metabolism, and migration.

## Discussion

Cannabis is the second most-smoked product after tobacco. As its use increases worldwide due to legalization and wider social acceptance, a growing number of users are exposed to thousands of potentially toxic combustions products. Given the hazards of smoking, users have sought other, safer methods of cannabis consumption, including the use of dry herb vaporizers that heat the cannabis flower without combustion. However, despite their heavy promotion as a possibly safer alternative, there exists no data on whether vaporizing cannabis affects the lungs. Inhaled compounds enter the lungs and reach the alveoli, which are the primary sites for gas exchange. As such, the alveoli are particularly vulnerable to the effects of airborne pollutants, irritants, and other toxicants. The large surface area of the alveoli facilitates the rapid absorption of inhaled chemicals into the bloodstream, including those that are contained within cannabis. However, alveoli are susceptible to damage by inhaled particles, and epithelial injury is linked to the mechanistic development of many lung diseases including chronic obstructive pulmonary disease, pulmonary fibrosis and lung cancer. Herein, we show that cannabis vapor is not inert; rather, it exerts significant effects on alveolar epithelial cells by changing the expression of genes involved in cell cycle control and metabolism.

One of our most concerning findings is the impact of cannabis vapor on genes associated with pathways related to cancer development in alveolar epithelial cells cultured at ALI. Lung cancer is a leading cause of cancer-related mortality worldwide and is caused largely by tobacco smoking (Thandra et al. 2021). Because both cigarette and cannabis smoke contain known carcinogens such as polycyclic aromatic hydrocarbons [PAHs], which are generated during the combustion process, there is concern that smoking cannabis will also lead to lung cancer development. Although the data is equivocal (Callaghan et al. 2013; Vásconez-González et al. 2023), this concern has at least partially driven the increase in popularity of dry herb vaporizers. Cannabis vapor is thought to have a reduced number and/or concentration of harmful combustion chemicals, leading to the assumption that inhalation of cannabis vapor is relatively risk-free. The data presented in this study cast doubt on this assumption, showing that cannabis vapor promotes the upregulation of genes within numerous cancer-related pathways.

Conserved changes across models show that cannabis vapor dysregulated pathways involved in cell cycle control and cellular metabolism. Our data using the TGx-DDI biomarker tool suggests that cannabis vaping is not DNA-reactive. However, carcinogens can also be non-genotoxic, promoting cancer through mechanisms that do not directly involve DNA damage, such as inflammation, immune system modulation, mitogenic signaling, and cell injury (Jacobs et al. 2020). It is noteworthy therefore that we see significant effects on genes that control many of these cellular mechanisms. Cannabis vapor increased genes related to mTOR signaling, such as *RICTOR* which promote cell growth and survival (O'Brien et al. 2011). *SMAD3* and *RUNX1*, which are involved in cell differentiation and growth control, were also induced (Lee et al. 2002). Furthermore, genes encoding proteins for cell adhesion, such as *GJA1* and *ITGB5*, were downregulated, suggesting a loss of cell-to-cell communication, which may contribute to cancer progression (James et al. 2018; Ojakian et al. 2001). Additionally, we observed a decrease in the tumor suppressor *DKK1*, which inhibits the Wnt/β-catenin signaling pathway that regulates cell proliferation and survival (Wall et al. 2020). Dysregulation of *DKK1* can promote tumor growth and metastasis, with suppression linked to more aggressive cancer phenotypes. Taken together, these changes can result in a microenvironment that favors uncontrolled cell proliferation and enhanced metastatic potential.

Cell proliferation relies on cellular metabolism to provide the energy and building blocks needed for cell cycle progression and rapid growth. In cells that have taken on a cancerous phenotype, metabolic pathways are reprogrammed to meet the heightened energy and biosynthetic demands of unchecked proliferation. Glycolysis becomes the primary source of energy production, even in the presence of oxygen (Warburg effect), while oxidative phosphorylation is often downregulated or modified (Pascale et al. 2020). Mitochondrial dysfunction, particularly through defects in the ETC, is closely linked to cancer development via mechanisms such as the Warburg effect. Our data show that cannabis vapor decreases the expression of genes encoding for ETC complex 1 ubiquinone oxidoreductase, complex III cytochrome c reductase, complex IV cytochrome c oxidase and complex V ATP synthase. This suggests that cannabis vapor causes deficiencies in the ETC which may contribute to increased production of ROS and mitochondrial DNA damage and can in turn activate oncogenic pathways and facilitate cancer progression and metastasis.

In addition to protein-coding genes, we also observed changes in ncRNAs including both lncRNA and miRNA, which can play crucial roles in cancer by acting within complex networks that affect gene expression and cellular pathways. As seen in Table 1, among the lncRNAs upregulated by cannabis vapor was LINC00115, which has emerged as a significant player in lung cancer. Research indicates that LINC00115 may function as a competitive endogenous RNA to modulate the activity of specific miRNAs, including miR-7. miR-7 regulates fibroblast growth factor 2 (FGF2), which can promote malignant transformation (Li et al. 2016). FGF2 is upregulated in lung cancer and is correlated with reduced patient survival (Li et al. 2016). Furthermore, LINC00115 may also promote the malignant properties of tumor cells by sponging miR-607, which affects the expression of integrin subunit beta 1 (ITGB1), a molecule critical in tumor growth and metastasis. The miRNAs upregulated by cannabis vapor are also involved in oncogenic pathways. miR-27b enhances cellular proliferation, inhibits apoptosis, and fosters migration and invasion, contributing to the cancer aggressiveness and spread (Liu et al. 2016). Another miRNA induced by cannabis vapor, MIR421, is markedly upregulated in NSCLC tissues and cell lines (Yang et al. 2019). Knockdown experiments demonstrate that reducing miR-421 levels can significantly inhibit NSCLC cell proliferation, induce cell cycle arrest, and decrease migratory and invasive capabilities (Yang et al. 2019). Lastly, tumor suppressor DIO3OS, which inhibits MYC activity, has been shown to be significantly downregulated in NSCLC (Zhang et al. 2021). Lower levels of DIO3OS correlate with worse patient outcomes, more aggressive cancer features, and reduced survival rates (Zhang et al. 2021).

Although cannabis vapor has fewer toxic byproducts, including carcinogens, compared to cannabis smoke, its effects on cellular health may arise from the presence of THC. Cannabinoids, including THC, can have oncogenic impacts on cells due to the central olivetol nucleus on their C-ring structure, a moiety implicated in damaging genetic material (Nahas et al. 1977; Reece and Hulse 2024). Exposure to cannabis and specific cannabinoids correlates with increased chromosomal anomaly rates and heightened cancer pathologies, even after rigorous adjustments for sociodemographic factors and reproductive outcomes (Albert Stuart and Gary 2016). Furthermore, THC has been shown to disrupt the microtubule machinery of the mitotic spindle, causing chromosomal abnormalities such as chromothripsis (Albert Stuart and Gary 2016). These abnormalities can activate oncogenes, silence tumor suppressor genes, and trigger other genotoxic events, underscoring the role of THC in promoting oncogenesis. As THC is the primary psychoactive component in cannabis, altering the exposure method of cannabis consumption cannot mitigate the adverse cellular impacts of this cannabinoid, emphasizing the need for further research into its health risks.

In this study, we modelled the AEC2 response to cannabis vapor using A549 cells, which are derived from a male with alveolar cell carcinoma (Swain et al. 2010). A549 cells retain many features of AEC2 cells, including synthesis of phospholipids, cytoplasmic lamellar bodies, and apical microvilli (Swain et al. 2010), making this a reasonable choice for this study. A limitation of using A549 cells is that they may not reflect all the biological outcomes of AEC2 cells. Our data shows that A549 cells cultured at ALI had increased expression of genes involved in surfactant production, ion transport, and barrier integrity, highlighting their increased ability to model the AEC2 phenotype. While an immortal cell line may not fully emulate the response of AEC2 cells to cannabis vapor, the use of in vitro preclinical models remains crucial to understand the cellular and molecular impact of inhaled exposures. The use of transformed cells can offer insights into novel mechanisms that would otherwise be difficult to ascertain, and a notable example is the discovery of NF-κB using human and mouse B cell lines (Sen and Baltimore 1986). Another limitation in the interpretation of the results is due to differences in cytotoxicity caused by vaporized cannabis between submerged and ALI culture protocols. This could explain the higher transcriptional response in A549 cells exposed to CaVE under traditional submerged conditions. Because we selected concentrations below which there was minimal cell death, we do not think this is the case. Moreover, vaporized cannabis caused significant changes in gene transcription and pathway enrichment irrespective of culture condition. By culturing these cell lines at ALI, we can elevate the relevancy of our preclinical findings. Nevertheless, our work represents the impacts of cannabis vapor on a single cell line; the lungs are a complex organ consisting of a myriad of cells that work together to respond to stimuli. Future work involving primary cells and animal models can help further elucidate the full spectra of impacts cannabis vapor poses to the pulmonary system.

In conclusion, our study is the first to examine the impact of vaporized cannabis on the transcriptome of alveolar epithelial cells. The upregulation of oncogenic pathways along with the downregulation of factors involved in tumorigenic suppression reinforces the need for comprehensive studies examining the health implications of emerging cannabis consumption methods, including vaporization, which is currently marketed as being safe. Understanding the extent to which inhalation of vaporized cannabis affects lung heath will be crucial for developing effective public health strategies and regulatory policies that mitigate the risks associated with cannabis use, particularly in jurisdictions where it has been legalized.

## Supplementary Information

Below is the link to the electronic supplementary material.

Supplementary file1 (DOCX 394 KB)

## Data Availability

All data are available within this article, online at https://figshare.com/s/9df93d5bda1d757f5cd7 and from the corresponding author upon reasonable request.
