# Supplementary material for: Transcriptomic changes in oxidative stress, immunity, and cancer pathways caused by cannabis vapor on alveolar epithelial cells
Source: Cell Biol Toxicol. 2025 Mar 8;41(1):57. doi: 10.1007/s10565-025-09997-3 (PMC11890392; doi:10.1007/s10565-025-09997-3)
Supplement: Supplementary file 1 — Supplementary file1 (DOCX 394 KB) [file 10565_2025_9997_MOESM1_ESM.docx]

**Online Supplement**

**Cell Biology and Toxicology**

**Transcriptomic** **Changes in Oxidative Stress, Immunity and Cancer Pathways Caused by Cannabis Vapor on Alveolar Epithelial Cells**

Emily T. Wilson^1,2^, Percival Graham^3^, David H. Eidelman^2,4^, and Carolyn J. Baglole^1,2,4*^

^1^Department of Pharmacology and Therapeutics, McGill University, Montreal, Quebec Canada

^2^Research Institute of the McGill University Heath Centre, Montreal Quebec Canada

^3^SCIREQ - Scientific Respiratory Equipment Inc., Montreal, Canada

^4^Department of Medicine, McGill University, Montreal, Canada

*Author for Correspondence:

Carolyn J. Baglole

Research Institute of the MUHC

Centre for Translational Biology (CTB), Block E

1001 Decarie Blvd.

Montreal QC H4A 3J1 Canada

Tel: (514) 934-1934 Ext 96109

Email: carolyn.baglole@mcgill.ca


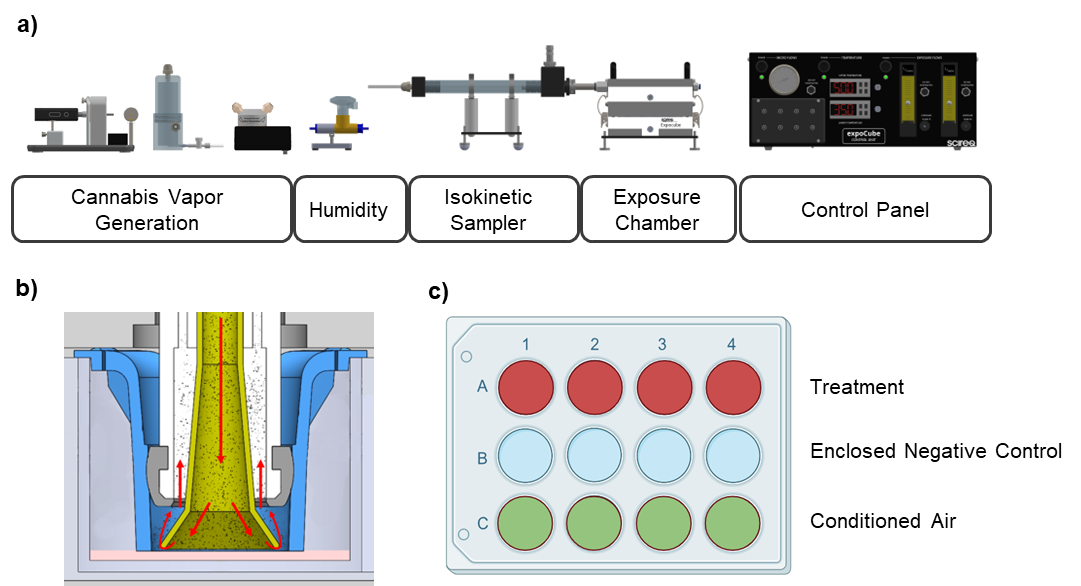


**Supplementary Fig. 1**. **Schematic of expoCube for Cannabis Vapor Exposure of ALI culture.** (a) DaVinci MIQRO vaporizer was used to generate cannabis vapor. This sample is humidified and shuttled through the isokinetic sampler and into the exposure site. The control unit allows adjustments to airflow outputs and temperature gradients. (b) Inside the well, trumpets deliver particles to the apical side of ALI cell cultures. (c) The expoCube is compartmentalized into three rows: a test row for cannabis vapor exposure, a vehicle control row of conditioned air, and the enclosed negative control row that remains untouched by any airflow.


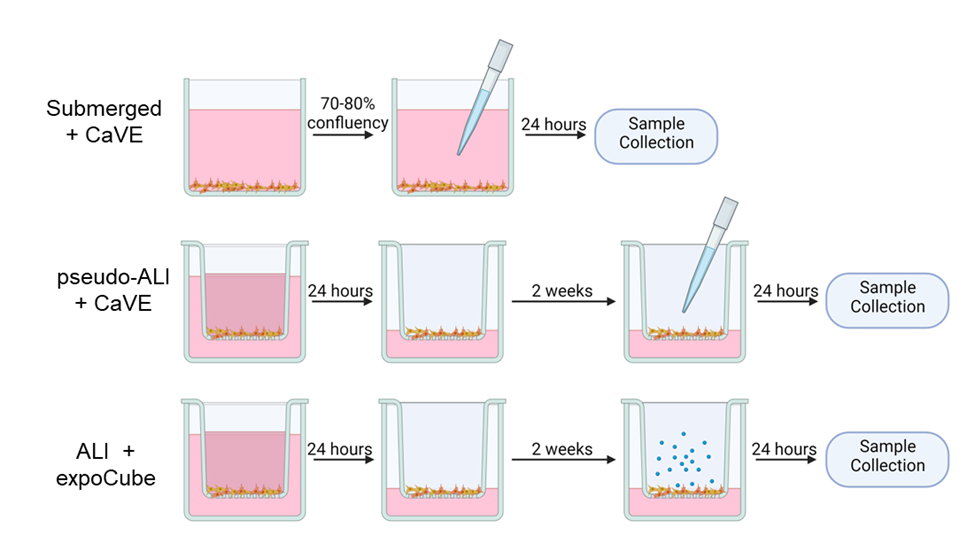


**Supplementary Fig. 2**. **Schematic of cell models and experimental outline** Experimental Design: Submerged model: A549 cells were grown in submerged conditions until reaching 70-80% confluency. They were then serum starved for 18 hours before exposure to CaVE. Samples were collected 24 hours post-treatment. Pseudo-ALI model: A549 cells were seeded into transwell inserts. After 24 hours the apical media was removed to create an ALI condition. Cells were kept at ALI for 2 weeks before treatment to allow for differentiation. Cells were serum starved 24 hours in advance of treatment. CaVE was added apically to cells dissolved in media for a total apical volume of 100 ul. Twenty-four hours after treatment samples were collected. ALI model: A549 cells were seeded into Transwell inserts. After 24 hours the apical media was removed to create an ALI condition. Cells were kept at ALI for 2 weeks before treatment to allow for differentiation. Cells were serum starved 24 hours in advance of treatment. Cells were exposed to cannabis vapor using the expoCube^TM^ for 30 minutes. Twenty-four hours after exposure samples were collected.
